# Nursing Institutions in the Country

**Published:** 1887-05-14

**Authors:** J. H. Timins

**Affiliations:** Founder of the Kent Nursing Institution


### Nursing Institutions in the Country.

By the Rev. J. H. Timins, M.A., F.G.S., Founder of the Kent Nursing Institution.

The excellent paper by Miss Manson, which was published in The Hospital on the 19th of February last, has met with the public attention which it so well deserved. Miss Manson, as the able Matron of St. Bartholomew's Hospital, treats the question from the hospital point of view, and writes of all nursing institutions unconnected with metropolitan hospitals in terms of general censure. But since the publication of her paper this has been qualified at The Hospitals Association meeting reported in The Hospital of February 26th; first, by Dr. Bedford Fenwick, who said that he "fully admitted that some nursing institutions did their work conscientiously and well"; and afterwards by the chairman of that meeting, Dr. G. W. Potter, who said that "there were nursing institutions now existing where really excellent nurses could be obtained, and that he wished to say that, in view of the somewhat indiscriminate charges made against existing nursing institutions." In fact, there are nursing institutions and nursing institutions. In London, as was to be expected from its great extent, may be found some of the best and some of the worst, with every intermediate variety of good and bad.

But it is not concerning them, but as regards nursing institutions in the country, that I now propose to write. I have been urged to do so by friends who think that my own extensive experience may enable me to give some useful information on the subject. That experience has been gained, first, by my having been, in my earlier days, a medical student, attending the practice of St. Thomas's Hospital for four consecutive years; and more recently by my having been concerned during the last twelve years in the conduct of the Kent Nursing Institution, which was founded by myself in the year 1875, for the purpose of supplying well-trained nurses, morally, intellectually, and professionally qualified for nursing rich and poor in the county of Kent.

The salaries of the nurses were originally fixed on a scale recommended for this institution by Miss Florence Nightingale. They are higher by one-fourth than those paid in any other nursing institution in the county; but Miss Nightingale considered that, having regard to the special qualifications required, they ought not to be less, and they have never been reduced. The nurses are also provided with uniform, which they always wear, indoors or out, excepting when away for a holiday; and they are all allowed to have a month's clear holiday in every year.

I will now consider the general subject of nursing institutions. Nurses are obtained for them, either by engaging nurses who have been already trained, or by having nurses trained for and at the cost of the institution, which is best. It is quite a mistake to suppose that "it is only now and again that a competent nurse enters a private institution." That will depend in a great measure upon the estimation in which an institution is held by nurses. Doubtless, as Miss Manson justly observes, "hospital authorities generally do their best to retain the services of their most competent nurses"; but that does not much affect the question. A nurse intended for private nursing may have too much of the hospital, as well as too little. I have not found that those who have been long hospital nurses are quite suitable for the more delicate work of private nursing. They are somewhat given to self-assertion, and deficient in tact, refinement, ready sympathy, and tender consideration for the feelings of their patients. They are easily disconcerted by cross accidents, and the difficulties of unexpected emergencies. They miss the conveniences of hospital organisation, and the supervision which saved them from the necessity of carrying their own brains, and are not fruitful in resources when left to shift for themselves. Nurses brought down by eminent London surgeons for operations in the country do not always leave by any means a favourable impression behind them. No doubt they are excellent nurses in point of skill; but private nursing is not quite in their line.

But apart from "hospital nurses" in the ordinary sense of the term, good nurses are undoubtedly to be found. I have read a letter from the matron of an institution containing as excellent nurses as any in England, in which she complained to our superintendent that "she was taking away all her best nurses." I have known very good nurses to leave a hospital or other institution for no other cause than a change of matron. Others have left to join friends in another home; others, for change from a northern to a southern climate; others, having relations dependent upon them, for an increase of professional income; others, it may be, in consequence of some little quarrel on a matter not affecting their general character or their reputation as nurses; whilst not a few have worked out their training at hospitals, intending all along to follow the profession of private nursing. Still, the utmost caution is needed in engaging nurses already trained; for there is a large amount of nursing rubbish in the market. But when the superintendent stands high, as she ought to do, in the nursing profession, and when the nurses' salaries are liberal, their treatment kind and generous, the nurses' home eligible in all respects, and the institution has a good reputation, the difficulty in obtaining the highest class of nurses is by no means insuperable.

But the best way of securing them is undoubtedly to have them trained; and provision ought to be made for annually training a number equal to one-seventh of that of the nursing staff required for the institution. The metropolitan hospitals have hitherto been medical schools for nurses as well as for doctors; and these schools are being one after another closed through hospitals starting private institutions.

The first sentence in Miss Manson's valuable paper is this: "Until recent times the great idea with regard to the sphere of the hospital seems to have been, not how far its useful influence and its work could be extended, but to what narrow limits it could be reduced." Surely something of this kind will be done by the hospital authorities themselves, if they close their hospitals as medical schools for the training of nurses for institutions all over England, in order that they may establish a monopoly in the business of private nursing for the metropolitan hospitals. It would be a great public advantage if they would lay down good rules for a course of efficient medical and surgical training for nurses, admit probationers only in accordance with those rules, give certificates only when the training should have been satisfactorily completed, and then allow those who were certificated the privilege of occasionally visiting their hospital to make themselves acquainted with any new methods and appliances connected with their profession.

I am glad to say that the London Hospital has not yet been affected by the prevailing institution mania. We have had several nurses trained there, and nothing could exceed the kindness and attention which the matron, Miss Lückes, has extended to our probationers, or the excellence of the training which she has given them.

As regards the length of time for which nurses should be trained, Miss Manson says: "The nurse who is intended for private work should receive at least two years' training at the expense of the hospital to which she is attached." But this opinion is not shared by all the managers of the metropolitan hospitals; for some of them are sending out nurses for private nursing who have only been one year in a hospital. In the country I only know of one institution, viz., the Kent Nursing Institution, to which I have before referred, and institutions connected with large provincial hospitals in which their own nurses are trained, as in Liverpool or Manchester, in which a full year's training in a first-class hospital is provided by them for their probationers. I can only name one, with the exceptions above named, and that in a northern county, in which the training is even as long as for nine months. The time for which nurses have been trained at London hospitals for other country institutions has been three or six months,—mostly, at the present time, six months, never more. Those institutions in which this is the maximum of hospital training have succeeded financially; from which it must be inferred that the nurses supplied by them came up to the requirements of those who have employed them, although the training is too short, and I may add that the salaries are too low, to secure any but such as Dr. Steele describes as "second-class nurses." But no one ought to be satisfied with only three months', or even six months' hospital training. It may do well enough for ordinary cases, which may be the majority; but it ought not to be considered sufficient for a nurse to succeed in nineteen cases out of twenty, if she be liable to fail through want of sufficient skill in the twentieth, when the sufferings of a patient might be increased, or a life endangered by it.

Miss Manson's able paper, and the important discussion to which it has given rise, will eventually have the effect of raising the standard of nursing efficiency throughout the country. In fact, it has already led people to investigate the claims of nurses to be called "hospital trained" in a way which they have never done before. But this will be a work of time. The supply will be regulated by the demand, and the want of highly trained nurses is not yet generally felt in the country.

Miss Manson says, that "when the nursing of a hospital is taught on the most thorough principle, the women who are attracted to it are of a class whose intelligence and education make them capable of being trained to a very high standard of efficiency"; and she seems to think that metropolitan hospitals have some exceptionally attractive power in this respect. But this is a gratuitous assumption. There are many such persons who would greatly prefer joining a well-conducted nursing institution, through which they will be received as pupils in a hospital in which they will be sure of receiving the best hospital training, than to join themselves as nurses to the hospital staff. As regards the admission and training of nurses in an institution, I will describe it as it is done in the Kent Nursing Institution, because it is the best known to me, and not at all assuming it to be better than what may be done in other nursing institutions.

Before anyone is admitted as a probationer, a full and searching inquiry is made as to her antecedents, family connections, education, moral and religious conduct, intelligence, ability, and constitution. If the result of this inquiry be in all respects satisfactory, the candidate is received into the Central Nurses' Home, where she remains for a time under the daily personal observation of the lady superintendent. If she be fully satisfied that the candidate has all the requisite qualifications for training as a thoroughly good nurse, she admits her as a probationer, and sends her for at least a year's training as a pupil in a first-class hospital, at the cost of the institution: keeping herself informed, by frequent communication with the training matron, as to the behaviour, industry, skill, and progress of her probationer.

After her year of training in medical and surgical nursing, if she be required for monthly nursing, she has to go to a leading lying-in hospital until she receive her certificate as a monthly nurse.

When a nurse returns to the home after the completion of her hospital training, she is instructed in the properties, use, and application of disinfectants, so as to enable her to guard successfully against the dissemination of any infectious disease, whether the isolation of her patient be practicable or not. I have fully explained how this can be done in my pamphlet on Artificial Disinfection (Churchill, 1878). The system has been adopted in other institutions in the home counties, and is now also taught in the Worcester Nursing Institution by the matron, Miss Holloway, who learnt it when she was a nurse in the Kent Nursing Institution, and who received the silver medal of the institution at the hands of the Duchess of Edinburgh, in recognition of her skill and self-devotion in suppressing a formidable outbreak of small-pox in Gravesend in the year 1879. No nurse's training ought to be considered complete until she has been thoroughly instructed in this very important work.

With regard to nurses' homes, I cordially agree with Miss Manson that "when not in charge of a case, the comfort of the nurses should in every way be provided for, so that their leisure time should be really a rest to them." Each nurse should have a separate bedroom or cubicle, and there should be beds enough for as many nurses as are at all likely to be at home at the same time, so that they should never be required to lodge out. Their home should be made comfortable in every way; there should be a good musical instrument in the nurse's own sitting-room, and a well-kept garden adjoining the house. There should also be a second house, with separate premises, and back and front entrance, disinfecting stoves, and every accommodation for a nurse in case of possible sickness, for the occupation of nurses who may have to be placed in quarantine. Their occasional seclusion will thus be made as little irksome and depressing as possible, and free from the painful feeling of isolation which they might have if sent away, entirely beyond the reach of the comforts, associations, and sympathies of home, to a distant lodging, or to a desolate ward at the top of a hospital.

When I say that all this should be done, I merely describe what is done, and what has been done for the last ten years for the nurses in the Kent Institution. And Miss Manson was led into a hasty generalisation when she said that "the nurses are more carefully selected, better supervised, and better cared for" in nursing homes which are connected with hospitals than in those which are not so connected.

